# Palaeoproteomics gives new insight into early southern African pastoralism

**DOI:** 10.1038/s41598-020-71374-3

**Published:** 2020-09-02

**Authors:** Louise Le Meillour, Séverine Zirah, Antoine Zazzo, Sophie Cersoy, Florent Détroit, Emma Imalwa, Matthieu Lebon, Alma Nankela, Olivier Tombret, David Pleurdeau, Joséphine Lesur

**Affiliations:** 1 UMR 7209 Archéozoologie, Archéobotanique: Sociétés, Pratiques et Environnements (AASPE), Muséum national d’Histoire naturelle, CNRS, 55 rue Buffon, 75005 Paris, France; 2 UMR 7245 Molécules de Communication et Adaptations des Microorganismes (MCAM), Muséum national d’Histoire naturelle, CNRS, 63 rue Buffon, 75005 Paris, France; 3 USR 3224 Centre de Recherche sur la Conservation (CRCC), Muséum national d’Histoire naturelle, CNRS, Ministère de la Culture, 36 rue Geoffroy Saint Hilaire, 75005 Paris, France; 4 UMR 7194 Histoire naturelle de l’Homme Préhistorique (HNHP), Muséum national d’Histoire naturelle, CNRS, UPVD, 1 rue René Panhard, 75013 Paris, France; 5 University of Namibia, Windhoek, Namibia; 6 National Heritage Council of Namibia, 153 Dr. AB May and Rev. Michael Scott streets, Ausspannplatz, Windhoek, Namibia

**Keywords:** Proteomics, Archaeology

## Abstract

The advent of domestication is a major step that transformed the subsistence strategies of past human societies. In Africa, domestic caprines (sheep and goat) were introduced in the north-eastern part of the continent from the Near East more than 9000 years ago. However, their diffusion southwards was slow. They are thought to have made their first appearance in the southern part of the continent *ca.* 2000 years ago, at a few Later Stone Age sites, including Leopard Cave (Erongo region, Namibia), which provided the oldest directly dated remains assigned to sheep or goat on the basis of morphology of bones and teeth. However, similarities in morphology, not only between these two domesticated caprine species, but also between them and the small wild antelopes, raised questions about the morphological species attribution of these remains. Additionally, the high fragmentation of the site’s osteological remains makes it difficult to achieve species-level taxonomic identification by comparative anatomy. In this paper, we report molecular species identification of the Leopard Cave remains using palaeoproteomics, a method that uses protein markers in bone and tooth collagen to achieve taxonomic identification of archaeological remains. We also report new direct radiocarbon dates. Wild antelope remains from museum collections were used to enrich the available protein record and propose de novo type I collagen sequences. Our results demonstrate that the remains morphologically described as domesticates actually belong to a wild antelope species and that domestic caprines first appeared at Leopard Cave 1500 years later than previously thought. This study illustrates that the use of palaeoproteomics coupled with direct radiocarbon dates is particularly suited to complement classic zooarchaeological studies, in this case concerning the arrival of the first herding practices in arid environments.

## Introduction

**Figure 1:**
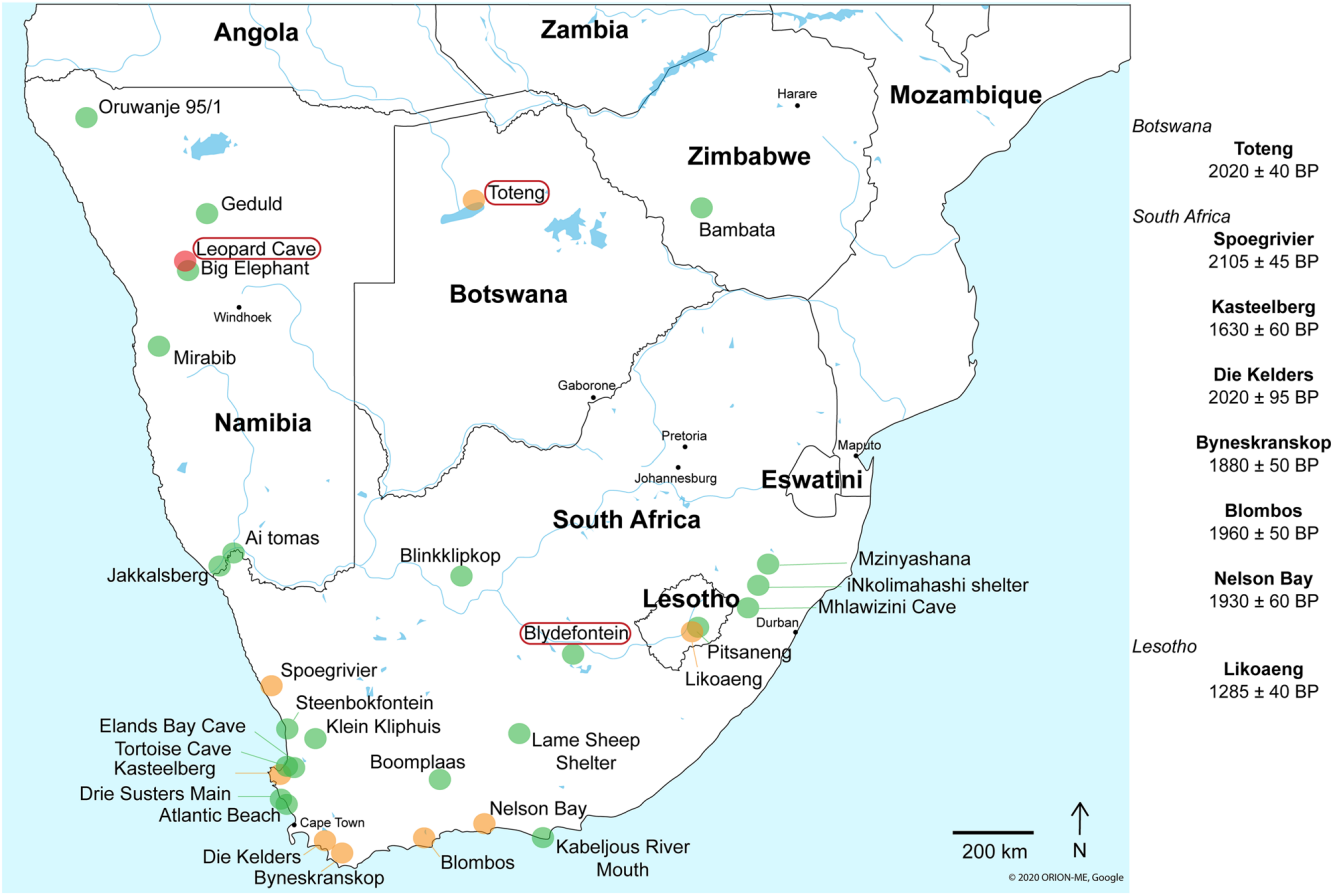
Map of southern Africa synthesizing all Later Stone Age sites from which caprine remains have been identified, including Leopard Cave (red dot), sites with remains attributed morphologically to caprines (after ref.^14^) (green dots), and sites with directly radiocarbon dated remains attributed morphologically to caprines (after ref.^15^) (orange dot). Site names circled in red refer to the three archaeological sites where caprine remains were molecularly identified (ref.^16^ and this study for Toteng and Leopard Cave; ref.^17^ for Blydefontein). Associated dates are given to the right of the map. The map was generated by Louise Le Meillour and David Pleurdeau using Google Maps (https://www.google.fr/maps) for country and river edges and created using Adobe Illustrator CC 2017 (https://www.adobe.com/).

Understanding how past human populations interacted with their environment, and particularly with other animals, allows understanding large parts of societies, their organisation and their economy. Climatic change is one of the factors that may have pressured human populations to adapt their subsistence strategy and diet. With Saharan and Sahelian aridification starting at the end of the African Humid Period (*ca.* 14,800–5500 years before present [BP]^1^), Africa experienced its last major climatic change^2^ and humans had to adapt to new environments^3^. Thus, the inception of domestication may have been constrained by fewer food resources and their increased unpredictability^4^. Given that they have no wild representatives on the continent, it is now commonly accepted that domestic sheep (*Ovis aries)* and goat (*Capra hircus*) were introduced from the Near East^5,6^. The first archaeozoological evidence of these species dates from the end of the 9th and the beginning of the 8th millennium BP, in the eastern part of the Sahara^7–13^. Yet, their diffusion across the continent was slow: the first domesticated caprines appear in southern Africa around 2000 BP (Fig. 1), and their presence is scarce within the faunal assemblages.

The modalities of the possible routes, timing, and diffusion of caprine herding practices are the subject of a long-standing debate amongst the scientific community^18–21^. Genetic^22–25^ and linguistic^26–28^ studies point toward an east African origin of the human populations and the herds, both of which could have reached the southernmost tip of the continent *ca.* 2300 years ago (see ref.^20,29^ for a recent review). The hypothesis of a migration of ‘proto-Khoekhoe’ populations is supported both by allelic similarities between Nama people from Namibia and Maasai people from east Africa^30–32^ and by the linguistic merging of click languages^28,33,34^. The presence of caprine species breeds (fat-tailed sheep, for example^35,36^) that are only represented in eastern and southern Africa is a supplementary argument in favour of strong networks of exchange between the two regions. Alternatively, the scarcity of remains of domesticated livestock in African Later Stone Age (LSA) archaeological sites has been argued to support the hypothesis of livestock diffusion from the northern part of Africa to the southern tip of the subcontinent by gradual infiltration, as has been demonstrated for other regions^37^. According to this model, domestic animals were introduced progressively into small, local hunter-gatherer groups through cultural percolation from livestock farmers from the north, implying a ‘low-intensity’ herding economy without any profound social or economic changes^20^. In this model, major cultural changes (technical or subsistence strategy shifts) that can be archaeologically attested arose considerably later and were linked to a more massive arrival of the Khoekhoe population, around 1000 BP^38^.

**Figure 2:**
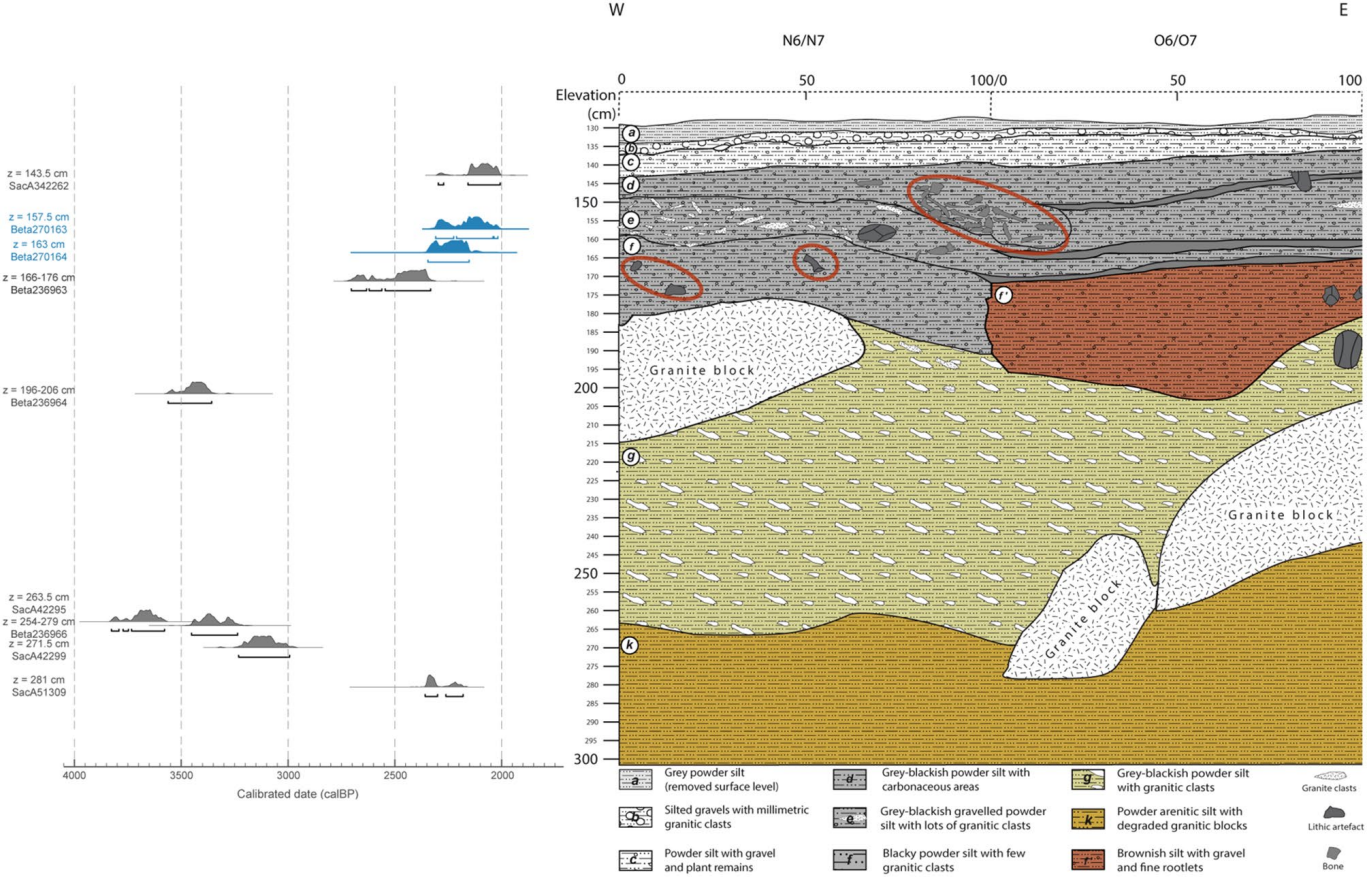
Stratigraphy of squares N6/N7 and O6/O7 in Leopard Cave. The stratigraphic position of the remains included in the study is indicated by red circles. All radiocarbon dates obtained within the sequence are presented by depth. The two dates corresponding to the direct dating of the teeth morphologically identified as caprine are shown in blue.

The western margins of the Kgalagadi basin and the Namib Desert, in Namibia, feature prominently in the question whether herding practices originated earlier, from the North, or later, from the South^19,39,40^. In central western Namibia, only a few archaeological sites have yielded early remains attributed to domesticated caprines, namely Geduld^41^, Mirabib^42^, Stripped Giraffe^43^, Oruwanje 95/1^44^ and Charè^45,46^. At the LSA site of Leopard Cave, in the Erongo region of the country^15^, remains identified morphologically as sheep or goat and directly radiocarbon dated (i.e. dated on the remains themselves) to 2300–2100 years cal. BP have been reported and thus were considered to be the oldest evidence of caprine herding. Extensive fieldwork has been ongoing at the site, mainly focused on the lower levels and rock art pigments^47^. In addition to the two dental remains previously described^15^, the excavations have yielded one mandible from the same layer and 25 remains from a layer higher up in the cave’s stratigraphic sequence (Fig. 2 and Table S1), all morphologically identified as possible caprines. The excavation campaigns also allowed refining of the chronostratigraphy of the rock shelter, confirming the integrity of the sequence, particularly for the upper layers, where the remains identified morphologically as caprines were recovered (Fig. 2 and Table S3).

Although the Leopard Cave remains fuelled a debate^14,18^ regarding the timing and process of the arrival of the first herding practices, the secure taxonomic identification of the first introduced caprine species in southern Africa (i.e. whether sheep or goat) is still pending^48–53^, even though nearly all the archaeological remains in this part of the continent have been attributed to *O. aries*^19^ based on comparative anatomy. Sheep and goat are morphologically close^54^; therefore, distinguishing between them is often a real challenge, especially given the high degree of bone fragmentation often observed in African archaeological contexts. The presence of mid-sized wild bovids that also share strong morphological similarities with domestic caprines makes morphological identification of bones and teeth particularly uncertain^49^. This problem is illustrated, for instance, by a recent morphological reassessment of a few bones of mid-sized bovid initially attributed to wild antelope species from the sites of Falls Rock Shelter and Snake Rock, on the Daures (also known as the Brandberg), Namibia, in layers dating from 2000 BP, which were reassigned to sheep^55^. Similarly, ancient DNA studies showed that remains morphologically identified as domestic caprines from Blydefontein rock shelter (South Africa) actually belong to several wild antelope species, including the springbok (*Antidorcas marsupialis*)^49^.

In this context, the use of molecular tools can be of great interest because ancient biomolecules preserved in archaeological remains can reveal features impossible to assess with traditional, morphological methods. In addition to ancient DNA studies, the past few years have witnessed a multiplicity of analyses based on the extraction and characterisation of long-term persistent biomolecules: proteins^56–61^. Since their amino acid sequence is coded for by the DNA, proteins carry genetically derived features that allow researchers to propose phylogenetic relationships of extinct species^62–65^, sex attribution^66,67^ or even characterisation of palaeopathogens^68^. The major protein of bone and teeth, type I collagen (constituting 90% of the organic phase of these materials), is composed of amino acid sequences that carry taxon-related differences in their order, allowing species discrimination^69^. Shifts in amino acids of type I collagen allow distinction between domestic caprine species, and palaeoproteomics is therefore particularly suited to the study of the diffusion of domesticates in southern Africa^16,57^. For the purpose of addressing this question in relation to Leopard Cave, we conducted palaeoproteomic analyses on all the remains morphologically identified as possible caprines among the mid-sized bovids, including ones identified below the taxonomic level of family.

## Results

Palaeoproteomic analyses were carried out on 28 remains from different layers of Leopard Cave. Two of them are dental remains from layer F identified as caprines^15^, one is a mandible half from layer E, and the other 25 are various skeletal elements from layer D (Fig. 2). The remains from layer E and D were morphologically identified as either caprines (sheep or goat) or mid-sized bovids (a category that includes sheep, goat, springbok and impala, *Aepyceros melampus*) (Table S1). Given the morphological proximity between these taxa and the absence of protein sequences available in the existing databases for mid-sized wild bovids, we simultaneously conducted proteomics on modern museum collection specimens of an Ethiopian sheep and a Namibian goat, as long as springbok and impala, two species which were present in the vicinity of Leopard Cave at the relevant time periods (Table S2).

### Identifying taxonomic markers

Proteomics analysis of the twentieth-century museum collection specimens of springbok and impala allowed us to reconstruct alpha 1 and alpha 2 chains of type I collagen (COL1A1 and COL1A2) using the PEAKS algorithm, which generates database-assisted de novo sequences (see Methods section). The generated type I collagen sequences of springbok and impala were aligned to available bovid reference sequences. The peptides observed in our dataset cover 97% and 67% of the reference mature COL1A1 sequence (i.e. the secreted protein, which only represents the helical regions of collagen) and 91% and 52% of the reference mature COL1A2 sequence for springbok and impala, respectively (Fig. S1 and Fig. S2). Sequence alignments point out several amino acid substitutions between the two species, but also with cattle (*Bos taurus*), sheep, goat and an Asiatic wild caprine, the chiru (*Pantholops hodgosonii)*. Several peptide taxonomic markers were thus identified: one on the alpha 1 chain and four on the alpha 2 chain of type I collagen (Table 1).

**Table 1:**
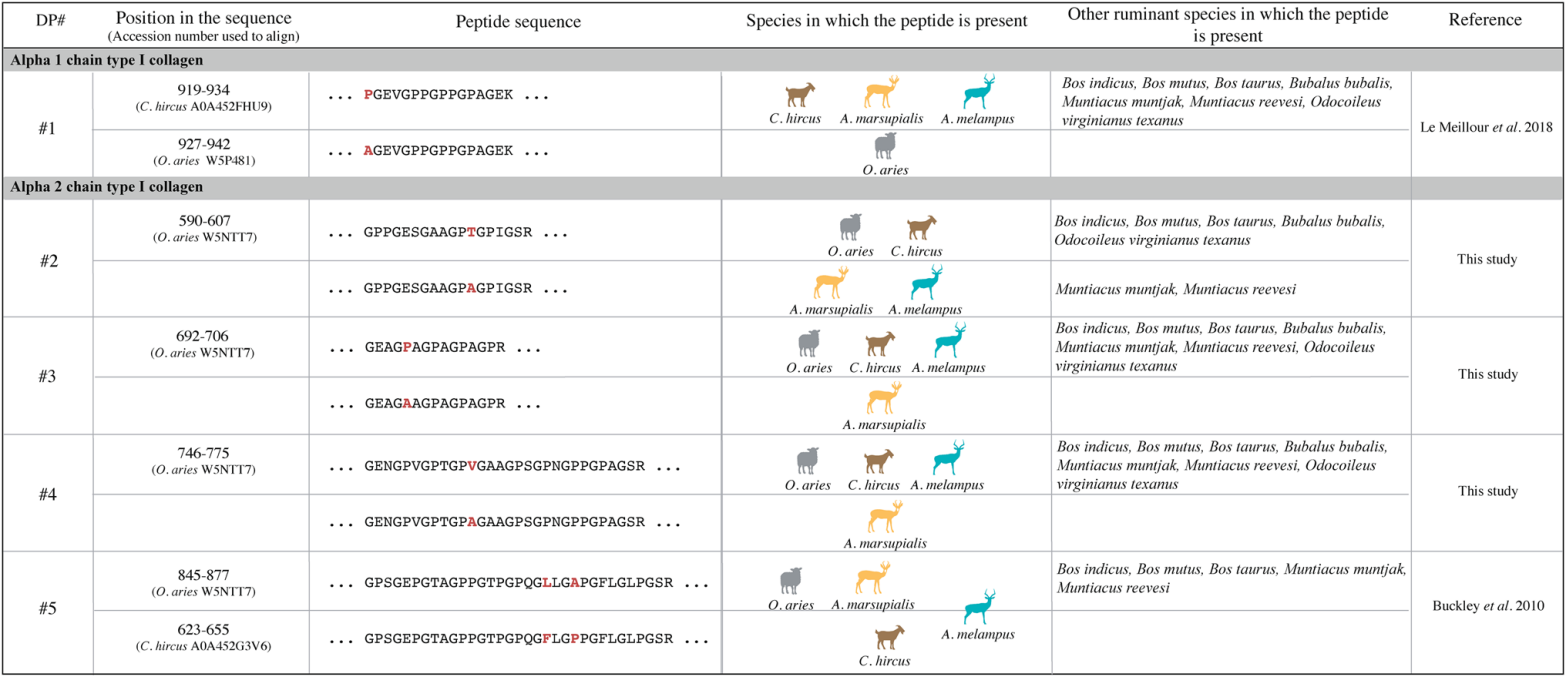
Alpha 1 and alpha 2 chains of type I collagen discriminating peptides of *Aepyceros melampus* and *Antidorcas marsupialis*.

Specific amino acids are represented in boldface red. Sheep is represented in grey; goat, in brown; springbok, in orange; and impala, in turquoise. Sequences were constructed using the PEAKS database-assisted de novo tool and aligned using Geneious software.

The sequences are written below, with specific amino acids in boldface and modified amino acids underlined (post-translational modifications). The springbok and the impala present one shared derived peptide on COL1A2, GPPGESGAAGP**A**GPIGSR (DP#2; Fig. S3a, Fig. S6). The springbok presents two uniquely derived peptides on COL1A2, that discriminates the species from the others bovids: GEAG**A**AGPAGPAGPR (DP#3; Fig. S3b, Fig. S6) and GENGPVGPTGP**A**GAAGPSGPNGPPGPAGSR (DP#4; Fig. S3c, Fig. S6). These three peptide markers allow the discrimination of springbok and impala from all other bovid species. In addition, our analyses indicate that the two peptide markers traditionally used for distinguishing sheep from goat on COL1A1^16^ and COL1A2^57,69,70^ are shared by the antelope species: the COL1A1 peptide **P**GEVGPPGPPGPAGEK (919–934, DP#1; Table S5) is shared between goat, springbok, and impala, and the COL1A2 peptide GPSGEPGTAGPPGTPGPQG**L**LG**A**PGFLGLPGSR (845–877, DP#5; Table S5) is shared between springbok and sheep (Table 1). For impala, we could not specify the sequence of DP#5, since both the sequences of sheep and goat were proposed as PSM (Table S5). The sequence PGEVGPPGPPGPAGEK was detected in almost every goat, impala and springbok sample, but always with at least one missed cleavage, contrary to the sheep sequence AGEVGPPGPPGPAGEK (Table S5). This is expected given the disfavoured proteolytic cleavage at the N-terminus of proline residues^71^.

### Taxonomic identification of archaeological samples and direct dating

The collagen content of the archaeological samples, measured using infrared spectroscopy (specifically, Fourier transform infrared spectroscopy in Attenuated Total Reflectance mode [ATR FT-IR]), varied between 11.2% and 22.1%weight (wt) of collagen preserved (Table S4). Compared with the modern bone value of 22%wt of collagen, the remains from Leopard Cave thus appear well to very well preserved; these values are certainly above the threshold of ~ 3%wt of collagen determined for radiocarbon and proteomics analysis^16,72^.

The protein extracts were analysed using liquid chromatography coupled to tandem mass spectrometry (a method known as ultra-high-performance liquid chromatography-tandem mass spectrometry [UHPLC-MS/MS]), and the data generated were examined for collagen correspondence through our local collagen sequence database, containing the de novo sequences of springbok and impala described above, and available collagen references of bovids (see Methods section for all accession numbers used). Protein extractions succeeded on each analysed remain selected for this study. Confidently identified peptides were assigned to alpha 1 and alpha 2 chains of type I collagen with sequence coverages of, on average, 70% of mature collagen for the 28 samples. Different algorithms were used, and raw data were manually assessed by extracting ion chromatograms and verifying fragment spectra of each taxonomic peptide reported for sheep and goat, as well as for the springbok and impala ones previously reported (Table S5).

Identifications were secured for all 28 of the remains by the presence of all the taxonomic markers of the corresponding species (Table 2). The three remains from layers E and F (LC_113, LC_149, and LC_176, respectively) present the COL1A1 peptide marker of goat and the COL1A2 marker of sheep, as well as the three new COL1A2 markers: the one shared between springbok and impala and the two others specific of springbok. The presence of the specific markers on the COL1A2 of springbok allows us to confidently identify the remains to this species (Table 2). One of the remains from layer D (LC_134) present a combination of COL1A1 and COL1A2 markers specific of the impala. The 24 other remains presented COL1A1 and COL1A2 peptide markers of sheep (Table 2, Table S5).

**Table 2:**
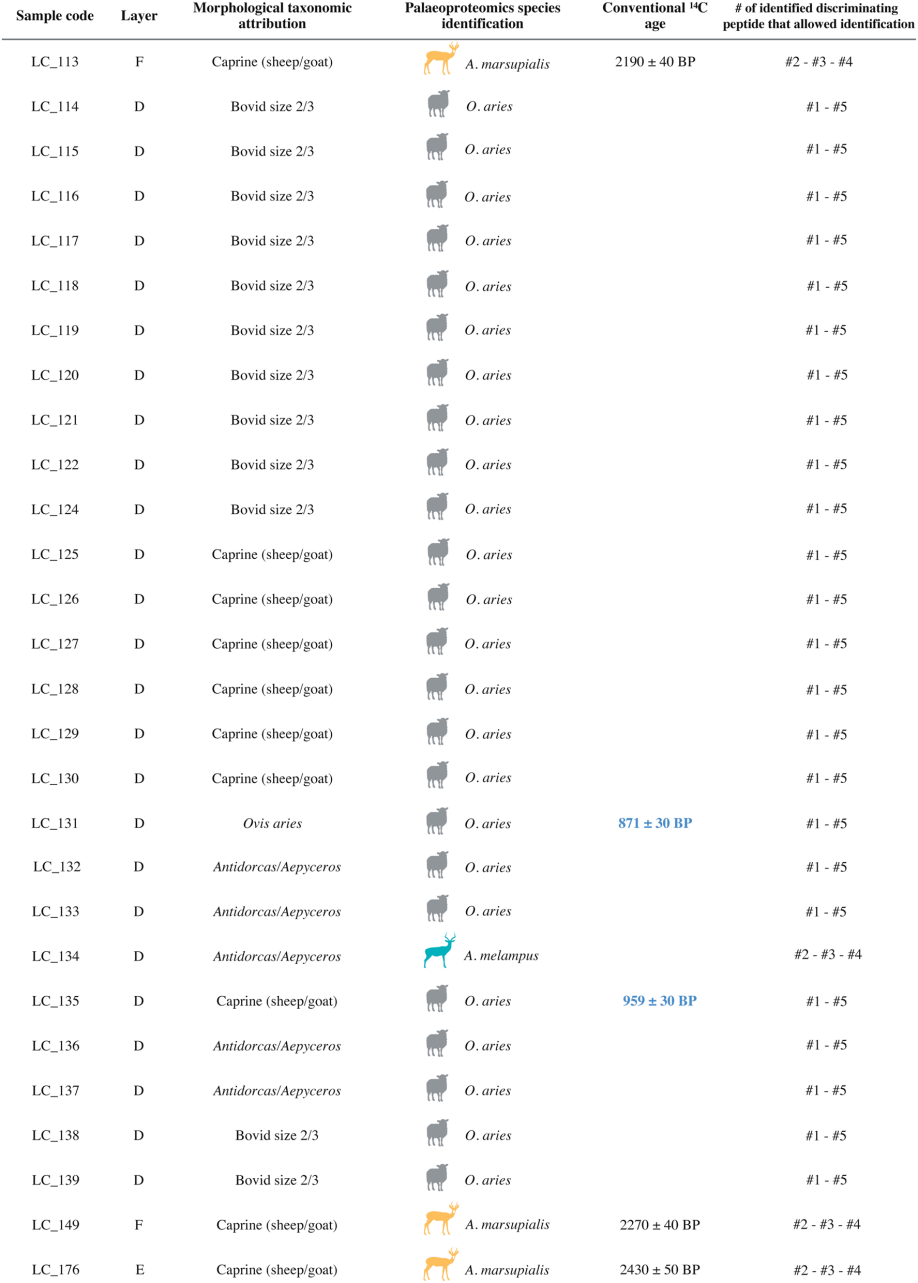
Species identifications of the 28 remains from Leopard Cave.

Each sample is identified by its lab code, the layer it was recovered from, the morphological identification, the molecular identification, and, if applicable, the corresponding radiocarbon date. The dates in boldface blue represent the direct dating for this study; the ones in black come from ref.^15^. The last column lists the discriminating peptide number present in the corresponding sample used for the species identification.

**Figure 3:**
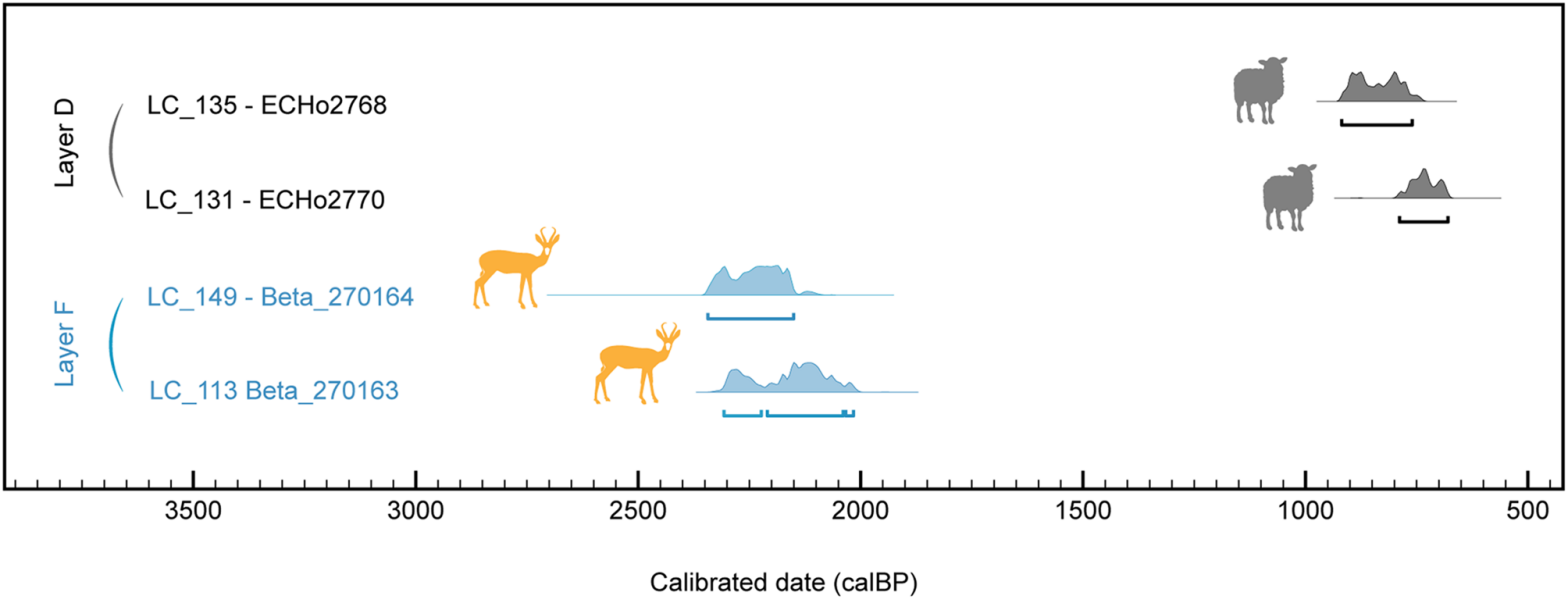
Leopard Cave mid-sized bovid remains direct radiocarbon dates. Direct dates previously reported for what are now known, based on proteomics, to be two springbok remains are shown in blue (LC_113, LC_149). New radiocarbon dates on remains identified as sheep by palaeoproteomics are shown in grey (LC_131, LC_135). Radiocarbon dates were calibrated at 2 σ (95.4%) using OxCal 4.3^73^ and the SHCal13^74^ calibration curve, for the southern hemisphere.

Two of the remains from layer D (LC_131 and LC_135) identified as sheep were selected for radiocarbon dating. These provided ^14^C ages of 870 ± 30 BP (789–680 cal. BP, 2σ) and 960 ± 30 BP (919–760 cal. BP, 2σ) (Fig. 3). These newly generated direct dating results make sense regarding the position of the dated samples within the stratigraphy (Fig. 2 and Table S3).

## Discussion

The proteomics analyses we performed allow us to propose de novo collagen sequences for two wild antelopes, the impala and the springbok, which were not yet available in the international databases. We identified three new markers on the alpha 2 chain of type I collagen that will be useful to identify archaeological remains of closely related mid-sized bovid species. We noticed that the markers of both of the type I collagen chains that are commonly used to discriminate between sheep and goat are shared by springbok and impala. In the case of the impala, we detected in our samples the COL1A2 marker DP#5 of both sheep/springbok and goat, suggesting that there might be an intra-specific variation of the peptide in this species. Since those peptides are widely used for species identification of archaeological remains^16,57,69,70^, we highly recommend the use of both of the type I collagen chain markers to assess attribution of bones and teeth to domestic caprines, especially in cases where the presence of wild antelopes within the archaeological assemblage is a possibility or already attested. All identified type I collagen peptide markers differ by one amino acid only between the different species, highlighting their phylogenetic proximity and the need to use multiple markers when working on closely related animal remains.

Based on the specific markers identified for springbok and impala and those previously reported in the literature for sheep and goat, we were able to disprove the original species attributions of the Leopard Cave remains dated to *ca*. 2300–2100 cal. BP. Our results suggest that domestic caprines were not present during this time range, and that they first appear in the cave sequence 1500 years later. We propose that environmental conditions could be one of the factors to explain this late presence of domesticates at Leopard Cave. Indeed, several authors have argued that the medieval warm period (occurring before the Little Ice Age) may have favoured increased precipitation in southern Africa between 1000 and 700 BP^75–77^. Wetter conditions may have created watering holes and improved grazing land availability and, thus, better environmental conditions for the management of livestock in the Namib region, particularly within each of the three inselbergs of Brandberg, Spitzkoppe, and Erongo. Remains from the LSA sites of Geduld^41^, Charè^45,46^, Mirabib^78^, Falls Rock, and Snake Rock^40^ may represent the earliest archaeological evidence of caprine introduction to what is now Namibia. However, none of these remains have been directly dated or molecularly analysed, raising questions about their antiquity and species determination. As our study illustrates, there is a need to be cautious under these circumstances. Thus, if collagen preservation of remains morphologically identified as domesticates allows it, and bearing in mind recent recommendations on the sampling of faunal remains^79^, we recommend the use of palaeoproteomics coupled with direct radiocarbon dating to document the early presence of caprines in Namibia and, more broadly, in southern Africa. This combination of methods will also help to accurately frame the chronology of the different waves of their introduction.

The archaeological evidence relating to pastoralism is thought to back up genetic and linguistic findings (specifically, the frequency of the lactase persistence allele among populations of ‘east Africans’ and the linguistic links between the Sandawe and KhoeKwadi languages among Khoekhoe populations)^24^. The low number of remains of domesticated caprines in LSA sites has been considered additional support for the hypothesis of the diffusion by gradual infiltration. In this model, the process of domestic caprines diffusion through Africa would have left no direct archaeological evidence of early herding practices. This ‘invisibility’ may also be due to the fact that populations exploited these domesticated animals mainly for their secondary products, such as milk or wool, or even for their carrying capacity, rather than their meat. Thus, the initial number of bones deposited and their degree of preservation, given variable taphonomic conditions, may influence the presence and detectability of domesticates in faunal assemblages. Hence, traces of herding practices may not be visible archaeologically, even though the animals being herded were eventually consumed by humans. Here again, the use of molecular methods would be appropriate, as the characterisation of pottery residues^80–82^ or dental calculus^83,84^ can reveal the exploitation of secondary products.

In addition, the identification of the first introduced sheep or goat is of particular importance to understanding the ecological, social, and subsistence behaviours of the first (semi-) pastoral societies. Because sheep and goat differ in their ability to adapt to climate change and harsh conditions^85^, people may have had a preference for one over the other when they first started exploiting domesticated caprines. Palaeoproteomics can thus play a leading role in the long-standing debate concerning the introduction of domestic sheep and/or goat throughout the subcontinent. This study particularly highlights the need to apply multiple lines of evidence when evaluating the modalities of the spread of pastoralism in southern Africa (and other parts of the continent), an application that is possible in the new era of biomolecular archaeology.

## Methods

We extracted and characterised ancient proteins from all the remains from the site morphologically attributed to caprines, *Antidorcas/Aepyceros* or mid-sized bovid, following recent recommendations on sampling and processing^79,80^. Having assessed their organic phase preservation using ATR FT-IR spectroscopy, we analysed ancient proteins from 25 bones and 3 dental remains (Table S1). In parallel, and based on the possibility of the presence of wild bovid species, we conducted proteomics analyses on molar roots of modern specimens of two wild antelopes (Artiodactyla, Bovidae), *Antidorcas marsupialis* (springbok, Antilopini) and *Aepyceros melampus* (impala, Aepycerotini) from the comparative anatomy collections of the Muséum national d’Histoire naturelle (Paris, France) (Table S2). One sheep and one goat remain from Namibia were also sampled for reference. We then radiocarbon dated two of the archaeological remains molecularly identified as domesticates, using accelerator mass spectrometry (AMS), in order to ascertain their antiquity.

### Archaeological remains from Leopard Cave

Leopard Cave is a rock shelter of approximately 50 square metres located in the locality of Omandumba West Farm, in the Erongo region of Namibia^15^. Archaeological excavations (2007–2019) have uncovered a long stratigraphic sequence, from the end of the Pleistocene to the Holocene (Fig. 1). The individual layers of the stratigraphic sequence have been previously published using numbers. Recent excavations (2017–2018) have allowed the refinement of the stratigraphy into three different complexes, and layers have now been redocumented by means of letter tags accordingly. Within a faunal assemblage largely dominated by wild game, only two dental remains in layer F (previously layer 6), one mandible in layer E (previously layer 5) and 25 remains in anatomical connection from an upper layer (layer D, previously layer 4) were identified morphologically as caprine or mid-size bovid (Table S1). They were all found in squares N6 and N7.

### Collagen preservation estimation

Collagen preservation was estimated prior to any palaeoproteomic analyses, in order to avoid unnecessary destruction of archaeological remains. Less than a milligram of bone or tooth powder was drilled and analysed using ATR FT-IR, following the method described for the radiocarbon sample selection^72^, and the exact same parameters were used to assess archaeological collagen preservation^16^.

### Protein extraction and treatment

All archaeological remains were sampled using a Dremel drill with a diamond bur that was rinsed with 96% EtOH between each sampling to avoid cross-contamination. A mean of 50 mg of bone or tooth powder was collected and chemically prepared following a modified version of the protocol previously published by our team for archaeological remains from African contexts^16^. In brief, samples (both modern and archaeological) were decalcified 8–15 days in a Tris–EDTA buffer (0.05 M and 0.5 M, final pH 7.4, Sigma Aldrich, St. Louis, Missouri, United States). Solutions were replaced every day and kept at 4 °C until further preparation (hereafter, decalcification solution, D). Once they were totally decalcified, pellets were rinsed with Milli-Q water until they were pH neutral. The remaining proteins were then resuspended in ammonium bicarbonate (ABC, 50 mM pH 8, Sigma Aldrich) for 3 h at 65 °C. After centrifugation, supernatants were collected (hereafter extract solutions, E) and placed into clean microtubes. D underwent buffer exchange from Tris–EDTA to ABC using 5 mL Vivaspin (3 kDa, Amicon Ultra, Merck Millipore, Burlington, Massachusetts, United States). Both D and E recovered proteins were reduced with dithiothreitol (DTT, final concentration of 10 mM, 20 min, 56 °C, 350 rpm), alkyled with iodoacetamide (10 mM, 30 min, in the dark, at room temperature) and subjected to trypsin hydrolysis (0.1 μg/μL, Trypsin Gold, Promega, Madison, Wisconsin, United States). Finally, D peptides were recovered, and solutions were desalted by solid phase extraction (SPE) using C8 cartridges (Sep-Pak C8 Plus Short 400 mg Sorbent, Waters, Milford, Massachusetts, United States). Protein concentrations were measured on a NanoVue instrument (GE HealthCare, Chicago, Illinois, United States), yielding amounts ranging between 0.54 and 1.48 μg/μL for archaeological samples and between 0.62 and 1.95 μg/μL for modern samples. Peptides in solutions were kept at − 20 °C until further analysis.

### Mass spectrometry analyses

The digested samples were analysed by ultra-high-performance-liquid-chromatography coupled to tandem mass spectrometry (UHPLC-MS/MS) following ref. ^16^. Briefly, we conducted the UHPLC separation on an Ultimate 3000-RSLC (Thermo Fisher Scientific, Waltham, Massachusetts, United States) using a RSLC Polar Advantage II Acclaim column (2.2 μm, 120 Å, 2.1 × 100 mm, Thermo Fisher Scientific). We used a mobile phase gradient composed of A: H_2_O + FA 0.1% and B: ACN + FA 0.08%, with a flow rate of 300 μL/min. The Electrospray-Quadrupole-Time of Flight (ESI-Q-TOF) mass spectrometer (Maxis II ETD, Bruker Daltonics, Billerica, Massachusetts, United States) was used in positive mode on the *m/z* range 200–2200 in data-dependent auto-MS/MS mode. MS/MS spectra were generated using collision-induced dissociation, with ion selection set to *m/z* 300–2200 and charge states set to 1^+^ to 5^+^. Data were automatically calibrated with sodium formate injected at the beginning of each analysis. An ABC blank was performed every five analyses to avoid cross-sample contamination.

The LC–MS/MS data were converted to .mgf format using DataAnalysis software (version 4.4, Bruker Daltonics). Sequences of the two wild antelopes species were reconstructed using the database-assisted de novo tool of PEAKS Studio software^86^ (version 7.5, BioInformatics Solutions, Waterloo, Canada). The search was conducted on a database combining the COL1A1 (*B. taurus* P02453, *B. indicus* A0A4W2FAL4, *B. mutus* L8IV51, *C. hircus* A0A452FHU9, *O. aries* W5P481, and *P. hodgosonii* XM_005964647.1) and COL1A2 (*B. taurus* P02465, *B. indicus* A0A4W2FTM9, *B. mutus* L8HQF7, *C. hircus* A0A452G3V6, *O. aries* W5NTT7, and *P. hodgosonii* XM_005985683.1) sequences of bovids. The entire PEAKS workflow was set to ‘trypsin’ enzymatic digestion, allowing 2 missed cleavages and including the following modifications: fixed carbamidomethylation (C) and variable oxidation (M), deamidation (NQ), hydroxylation (P) and phosphorylation (ST). Error tolerance was set to 10 ppm for precursors and 0.02 Da for fragment ions. Both SPIDER and PEAKS DB tools searches were used to propose the new COL1A1 and COL1A2 sequences. Each amino acid substitution proposed by the software was verified manually by looking at the MS/MS spectra, using the reference sequence that allowed the best coverage percentage. The de novo scores were set to 1% FDR, score of 50% and protein hit ≥ 20. The combined Mascot protein results were generated with ProteinScape Version 4.0.2 (Bruker Daltonics), using the Bovidae collagen database used in PEAKS, complemented by the impala and springbok sequences generated de novo. Once confidently identified, the COL1A1 and COL1A2 were aligned using Geneious^87^ software (version 11.0.3, Auckland, New Zealand; substitution matrix Blosum62, gap open penalty 12) to corresponding protein sequences of *B. taurus* (NM_001034039.2 and NM_174520.2), *B. indicus* (XM_019981159.1 and XM_019959690.1), *B. mutus* (XM_005890325.2 and XM_005909695), *C. hircus* (XM_018064893.1, XM_018064894.1 and XM_0056778936.3), *O. aries* (XM_015098715.1 and XM_004007726.3), *O. aries musimon* (XM_012127623.2, XM_012127624.2 and XM_012146567.2) and *P. hodgosonii* (XM_005964647.1 and XM_005985683.1).

Archaeological samples digests data were searched against a restricted protein database using Mascot software (version 2.4.1, www.matrixscience.com, London, UK^88^). The database was assembled using the National Center for Biotechnology Information (NCBI) collagen sequences (last updated December 9, 2018; 88530 sequences for 78817228 residues, all entries). The same set of parameters used for the PEAKS search was used for the Mascot search. Since the results did not allow to propose clear species identification, they were analysed using ProteinScape software (Bruker, Mannheim, Germany) by implementing the new COL1A1 and COL1A2 sequences of springbok and impala previously de novo reconstructed to the bovid species type I collagen sequence databases. Note that we cannot rule out Leu/Ile substitution in the marker sequences as standard MS/MS cannot differentiate between these two isomeric amino acids.

### Radiocarbon dating

We immersed coarse bone powder (0.3–0.7 mm) in 1 M HCl for 20 min under continuous stirring. We then separated the acid-insoluble residues from the solution by centrifugation and rinsed them with Milli-Q water, using vortex. We then immersed them in 0.1 M NaOH for 30 min under stirring, with a change of solution after 10 min. After a new rinsing with Milli-Q water using centrifugation, we immersed the alkali-insoluble residues in 0.01 M HCl and performed solubilisation at 95 °C overnight. We filtered the final solution on MF-Millipore membranes (mixed cellulose ester membranes of 5.0 µm pore size from Fisher Scientific, Illkirch, France) before freeze-drying them and collecting them for analysis. We performed sample graphitization using the automated AGE 3 graphitization unit and AMS measurements using the compact AMS ECHoMICADAS at the Laboratoire des Sciences du Climat et de l’Environnement (LSCE, CEA, CNRS, UVSQ).

### Data availability

The mass spectrometry proteomics data have been deposited to the ProteomeXchange Consortium via the PRIDE^89^ partner repository, with the dataset identifier PXD017519 and 10.6019/PXD017519. Each file name in ProteomeXchange corresponds to the nomenclature used for the sample’s code, both in this paper and in the associated supplementary data. Sequences of type I collagen for springbok and impala have been deposited on the UniProt database.

## Supplementary information

Supplementary Information.

Supplementary Table.
