# Supplementary material for: Palaeoproteomics gives new insight into early southern African pastoralism
Source: Sci Rep. 2020 Sep 2;10:14427. doi: 10.1038/s41598-020-71374-3 (PMC7468255; doi:10.1038/s41598-020-71374-3)
Supplement: Supplementary file 1 — Supplementary Information. [file 41598_2020_71374_MOESM1_ESM.pdf]

Palaeoproteomics gives new insight into early southern African pastoralism

Louise Le Meillour, Séverine Zirah, Antoine Zazzo, Sophie Cersoy, Florent Détroit, Emma Imalwa, Matthieu Lebon, Alma Nankela, Olivier Tombret, David Pleurdeau & Joséphine Lesur

Supplementary material

Archaeological remains from Leopard Cave included in this study. Previously published (Pleurdeau *et al*. 2012) and new radiocarbon dates (LC_131 and 135) were calibrated at 2 σ (95.4%), using OxCal 4.3 online (Bronk Ramsey 2017) and the SHCal13 calibration curve (Hogg *et al*. 2013). **Supplementary Table S1** *3*

Modern samples from the comparative anatomy collections of the Muséum national d'Histoire naturelle, Paris, included in this study. **Supplementary Table S2** *4*

Radiocarbon dates obtained on samples from the Leopard Cave stratigraphy. Dates are presented in the sequence of their depth within the archaeological sequence. Dates were calibrated at 2 σ (95.4%), using OxCal 4.3 (Bronk Ramsey 2017) and the SHCal13 calibration curve (Hogg *et al*. 2013). **Supplementary Table S3** *5*

Organic phase preservation estimation of Leopard Cave remains by ATR FT-IR. **Supplementary Table S4** *6*

List of sheep, goat, springbok and impala markers detected in the modern reference samples and archaeological samples from Leopard Cave. Colors correspond to molecular identification. Grey indicates peptides specific to sheep; brown, to goat; yellow, to springbok; and turquoise, to impala. Sheet 1: peptides detected in the modern reference samples. Sheet 2: peptides detected in the archaeological samples from Leopard Cave. Sheet 3: Number of peptide spectrum match (PSM) per sample. Numbers in red correspond to low confident MS/MS spectrum of the corresponding peptide. **Supplementary Table S5** ***see file TableS5.xls***

Number of queries and of peptide spectral matches (PSMs) for each archaeological sample. **Supplementary Table S6.** *14*

Sequence coverages of the alpha 1 and 2 type I collagen helical regions (ref. *C. hircus* for COL1A1 and *O. aries* for COL1A2) of the springbok. Grey indicates coverage of the reference sequence. Red indicates coverage of the springbok-specific peptides. **Supplementary Fig. S1** *7*

Sequence coverages of the alpha 1 and 2 type I collagen helical regions (ref. *C. hircus* for COL1A1 and *O. aries* for COL1A2) of the impala. Grey indicates coverage of the reference sequence. Red indicates coverage of the impala-specific peptides. **Supplementary Fig. S2** *8*

MS/MS spectra of distinguishing peptides of springbok and impala obtained as a result of *de novo* analyses of modern reference samples from the comparative anatomy collections of the Muséum national d'Histoire naturelle, Paris. **Supplementary Fig. S3** *9-10*

MS/MS spectra of distinguishing peptides for archaeological samples LC_113 (A); LC_149 (B); and LC_176 (C). **Supplementary Fig. S4** *11-13*

Supplementary Table S1. Archaeological remains from Leopard Cave included in this study. Previously published (Pleurdeau *et al*. 2012) and new radiocarbon dates (LC_131 and 135) were calibrated at 2 σ (95.4%), using OxCal 4.3 online (Bronk Ramsey 2017) and the SHCal13 calibration curve (Hogg *et al*. 2013).

Sample code	Stratigraphic position details	Morphological taxonomic attribution	Anatomical element	Biomolecular identification	Conventional ^{14}C age	Calibrated ^{14}C age (2σ)	Reference
LC_113	157,5 cm - Square N7 - Layer F	Caprine (sheep/goat)	Lower right M3	*Antidorcas marsupialis*	2190 ± 40 BP	358–67 cal. BCE	Beta270163, Pleurdeau *et al*. 2012
LC_114	OS CONX 1 - 146–156 cm - Square N7 - Layer D	Bovid size 2/3	Metacarpal	*Ovis aries*			This paper
LC_115	OS CONX 1 - 146–156 cm - Square N7 - Layer D	Bovid size 2/3	Metacarpal	*Ovis aries*			This paper
LC_116	OS CONX 1 - 146–156 cm - Square N7 - Layer D	Bovid size 2/3	Phalanx 1	*Ovis aries*			This paper
LC_117	OS CONX 1 - 146–156 cm - Square N7 - Layer D	Bovid size 2/3	Phalanx 1	*Ovis aries*			This paper
LC_118	OS CONX 1 - 146–156 cm - Square N7 - Layer D	Bovid size 2/3	Phalanx 1	*Ovis aries*			This paper
LC_119	OS CONX 1 - 146–156 cm - Square N7 - Layer D	Bovid size 2/3	Phalanx 1	*Ovis aries*			This paper
LC_120	OS CONX 1 - 146–156 cm - Square N7 - Layer D	Bovid size 2/3	Phalanx 1	*Ovis aries*			This paper
LC_121	OS CONX 1 - 146–156 cm - Square N7 - Layer D	Bovid size 2/3	Phalanx 1	*Ovis aries*			This paper
LC_122	OS CONX 1 - 146–156 cm - Square N7 - Layer D	Bovid size 2/3	Phalanx 1	*Ovis aries*			This paper
LC_124	OS CONX 1 - 146–156 cm - Square N7 - Layer D	Bovid size 2/3	Phalanx 2	*Ovis aries*			This paper
LC_125	OS CONX 1 - 146–156 cm - Square N7 - Layer D	Caprine (sheep/goat)	Metacarpal	*Ovis aries*			This paper
LC_126	OS CONX 1 - 146–156 cm - Square N7 - Layer D	Caprine (sheep/goat)	Phalanx 2	*Ovis aries*			This paper
LC_127	OS CONX 1 - 146–156 cm - Square N7 - Layer D	Caprine (sheep/goat)	Phalanx 2	*Ovis aries*			This paper
LC_128	OS CONX 1 - 146–156 cm - Square N7 - Layer D	Caprine (sheep/goat)	Phalanx 3	*Ovis aries*			This paper
LC_129	OS CONX 1 - 146–156 cm - Square N7 - Layer D	Caprine (sheep/goat)	Phalanx 3	*Ovis aries*			This paper
LC_130	OS CONX 1 - 146–156 cm - Square N7 - Layer D	Caprine (sheep/goat)	Phalanx 3	*Ovis aries*			This paper
LC_131	OS CONX 1 - 146–156 cm - Square N7 - Layer D	*Ovis aries*	Talus	*Ovis aries*	871 ± 30 BP	1162–1271 cal. CE	ECHo_2770, this paper
LC_132	OS CONX 1 - 146–156 cm - Square N7 - Layer D	*Antidorcas*/*Aepyceros*	Phalanx 2	*Ovis aries*			This paper
LC_133	OS CONX 1 - 146–156 cm - Square N7 - Layer D	*Antidorcas*/*Aepyceros*	Phalanx 2	*Ovis aries*			This paper
LC_134	OS CONX 1 - 146–156 cm - Square N7 - Layer D	*Antidorcas*/*Aepyceros*	Phalanx 3	*Ovis aries*			This paper
LC_135	OS CONX 2 - 146–156 cm - Square N7 - Layer D	Caprine (sheep/goat)	Phalanx 3	*Ovis aries*	959 ± 30 BP	1032–1190 cal. CE	ECHo_2768, this paper
LC_136	146–156 cm - Square N7 - Layer D - n° 69	*Antidorcas*/*Aepyceros*	Coxal	*Ovis aries*			This paper
LC_137	146–156 cm - Square N7 - Layer D - n° 73	*Antidorcas*/*Aepyceros*	Tibia	*Ovis aries*			This paper
LC_138	146–156 cm - Square N7 - Layer D - n° 80	Bovid size 2/3	Tibia	*Ovis aries*			This paper
LC_139	146–156 cm - Square N7 - Layer D - n° 89	Bovid size 2/3	Maxilla	*Ovis aries*			This paper
LC_149	166 cm - Square N7 - Layer F	Caprine (sheep/goat)	Upper right M2	*Antidorcas marsupialis*	2270 ± 40 BP	394–202 cal. BCE	Beta270164, Pleurdeau *et al*. 2012
LC_176	163 cm - Square N6 - Layer E	Caprine (sheep/goat)	Right hemi-mandible	*Antidorcas marsupialis*	2430 ± 50 BP	753–382 cal. BCE	Charcoal in same layer, Beta236963, Pleurdeau *et al*. 2012

Supplementary Table S2. Modern samples from the comparative anatomy collections of the Muséum national d'Histoire naturelle, Paris, included in this study.

**Sample code**	**MNHN inventory number**	**Provenance**	**Taxonomic identification**	**Anatomical part**
IMP 118	MNHN_ZM_AC_1945-118	Unknown	*Aepyceros melampus*	Lower M2 (root)
IMP 119	MNHN_ZM_AC_1945-119	Unknown	*Aepyceros melampus*	Upper P3 (root)
SPR 89	MNHN_ZM_AC_1971-89	Ménagerie du Jardin des Plantes, Paris	*Antidorcas marsupialis*	Upper M2 (root)
SPR 1670	MNHN_ZM_AC_1993-1670	Parc zoologique de Paris	*Antidorcas marsupialis*	Upper P4 (root)
CHE_06	NAM_CHE_2016_06	Namibia	*Capra hircus*	Upper right M2 (root)
ABY	MNHN_ZM_AC_1886-383	Ethiopia	*Ovis aries*	Upper P4

Supplementary Table S3. Radiocarbon dates obtained on samples from the Leopard Cave stratigraphy. Dates are presented in the sequence of their depth within the archaeological sequence. Dates were calibrated at 2 σ (95.4%), using OxCal 4.3 (Bronk Ramsey 2017) and the SHCal13 calibration curve (Hogg *et al*. 2013).

Lab#	Year	Square	Depth (cm)	Material	Conventional radiocarbon age	Calibrated age (2 σ)
SacA342262	2013	N6	143.5	Charcoal	2150 ± 30 BP	2295–2005 cal BP
Beta270163	2010	N7	157.5	Bovid tooth	2190 ± 40 BP	2307–2016 cal BP
Beta270164	2010	N7	163	Bovid tooth	2270 ± 40 BP	2343–2151 cal BP
Beta236963	2009	N7	166-176	Charcoal	2430 ± 50 BP	2702–2331 cal BP
Beta236964	2009	N7	196-206	Charcoal	3250 ± 40 BP	3560–3356 cal BP
Beta236966	2009	N7	254-279	Charcoal	3180 ± 40 BP	3450–3235 cal BP
SacA42295	2015	N7	263.5	Charcoal	3465 ± 30 BP	3825–3577 cal BP
SacA42299	2015	N7	271.5	Charcoal	3000 ± 30 BP	3230–2991 cal BP
SacA51309	2017	N7	281	Charcoal	2330 ± 30 BP	2357–2179 cal BP

Supplementary Table S4. Organic phase preservation estimation of Leopard Cave remains by ATR FT-IR.

Sample code	AmideI/PO4	%N wt	%wt collagen
LC_113	0.084	2.050	11.240
LC_114	0.113	2.640	14.485
LC_115	0.143	3.257	17.876
LC_116	0.126	2.902	15.924
LC_117	0.125	2.875	15.777
LC_118	0.128	2.944	16.154
LC_119	0.120	2.784	15.275
LC_120	0.140	3.188	17.493
LC_121	0.158	3.566	19.570
LC_122	0.127	2.930	16.080
LC_124	0.158	3.556	19.518
LC_125	0.157	3.545	19.455
LC_126	0.139	3.178	17.440
LC_127	0.160	3.604	19.780
LC_128	0.167	3.755	20.612
LC_129	0.170	3.817	20.947
LC_130	0.143	3.251	17.842
LC_131	0.120	2.780	15.255
LC_132	0.139	3.164	17.364
LC_133	0.145	3.294	18.079
LC_134	0.162	3.645	20.003
LC_135	0.156	3.522	19.328
LC_136	0.168	3.780	20.744
LC_137	0.142	3.231	17.729
LC_138	0.122	2.830	15.532
LC_139	0.180	4.024	22.086
LC_149	0.127	2.930	16.100
LC_176	0.104	2.450	13.450
Modern_ref_*B. taurus*	0.163	3.680	20.170

Supplementary Fig. S1. Sequence coverages of the alpha 1 and 2 type I collagen helical regions (ref. *C. hircus* for COL1A1 and *O. aries* for COL1A2) of the springbok. Grey indicates coverage of the reference sequence. Red indicates coverage of the springbok-specific peptides.

>Alpha 1 chain collagen type I [*Antidorcas marsupialis*] 97% coverage *C. hircus*

```
QLSYGYDEKSTGISVPGPMGPSGPRGLPGPPGAPGPQGFQGPPGEPGEPGASGPMGPRGPPGPPGKNG
DDGEAGKPGRPGERGPPGPQGARGLPGTAGLPGMKGHRGFSGLDGAKGDAGPAGPKGEPGSPGENGAP
GQMGPRXXXXXXGRPGAPGPAGARGNDGATGAAGPPGPTGPAGPPGFPGAVGAKGEAGPQGPRGSEGP
QGVRGEPGPPGPAGAAGPAGNPGADGQPGAKGANGAPGIAGAPGFPGARGPSGPQGPSGPPGPKGNSG
EPGAPGSKGDTGAKGEPGPTGIQGPPGPAGEEGKRGARGEPGPAGLPGPPGERXXXXXXGFPGSDGVA
GPKGPAGERGAPGPAGPKGSPGEAGRPGEAGLPGAKGLTGSPGSPGPDGKTGPPGPAGQDGRPGPPGP
PGARGQAGVMGFPGPKGAAGEPGKXXXXGVPGPPGAVGPAGKDGEAGAQGPPGPAGPAGERGEQGPAG
SPGFQGLPGPAGPPGEAGKPGEQGVPGDLGAPGPSGARXXXXXXXXXGVQGPPGPAGPRGANGAPGND
GAKGDAGAPGAPGSQGAPGLQGMPGERGAAGLPGPKGDRGDAGPKGADGAPGKDGVRGLTGPIGPPGP
AGAPGDKGETGPSGPAGPTGARGAPGDRGEPGPPGPAGFAGPPGADGQPGAKGEPGDAGAKGDAGPPG
PAGPAGPPGPIGNVGAPGPKGARGSAGPPGATGFPGAAGRVGPPGPSGNAGPPGPPGPAGKEGSKGPR
GETGPAGRPGEVGPPGPPGPAGEKGAPGADGPAGAPGTPGPQGIAGQRGVVGLPGQRGERGFPGLPGP
SGEPGKQGPSGASGERGPPGPMGPPGLAGPPGESGREGAPGAEGSPGRDGAPGAKGDRGETGPAGPPG
APGAPGAPGPVGPAGKSGDRGETGPAGPAGPIGPVGARGPAGPQGPRGDKGETGEQGDRGIKGHRGFS
GLQGPPGPPGSPGEQGPSGASGPAGPRGPPGSAGTPGKDGLNGLPGPIGPPGPRGRTGDAGPAGPPGP
PGPPGPPGPPSGGYDLSFLPQPPQEKXXXXXXXXXX
```

>Alpha 2 chain collagen type I [*Antidorcas marsupialis*] 91% *O. aries*

```
QFDGKGGGPGPMGLMGPRGPPGASGAPGPQGFQGPPGEPGEPGQTGPAGARXXXXXXXXAGEDGHPGK
PGRPGERGVVGPQGARGFPGTPGLPGFKGIRGHNGLDGLKGQPGAPGVKGEPGAPGENGTPGQTGARX
XXXXXGRVGAPGPAGARGSDGSVGPVGPAGPIGSAGPPGFPGAPGPKGELGPVGNPGPAGPAGPRGEV
GLPGLSGPVGPPGNPGANGLPGAKGAAGLPGVAGAPGLPGPRGIPGPVGAAGATGARGLVGEPGPAGS
KGESGNKGEPGAVGQPGPPGPSGEEGKRGSTGEIGPAGPPGPPGLRGNPGSRGLPGADGRAGVMGPAG
SRGATGPAGVRGPNGDSGRPGEPGLMGPRGFPGSPGNIGPAGKEGPAGLPGIDGRPGPIGPAGARGEP
GNIGFPGPKGPTGDPGKAGEKGHAGLAGPRGAPGPDGNNGAQGPPGLQGVQGGKGEQGPAGPPGFQGL
PGPAGTAGEAGKPGERGIPGEFGLPGPAGARGERGPPGESGAAGPAGPIGSRGPSGPPGPDGNKGEPG
VVGAPGTAGPSGPSXXXXXXGAAGIPGGKGEKGETGLRGDVGSPGRDGARGAPGAVGAPGPAGANGDR
GEAGAAGPAGPAGPRGSPGERGEVGPAGPNGFAGPAGAAGQPGAKGERXXXGPKGENGPVGPTGPAGA
AGPSGPNGPPGPAGSRGDGGPPGATGFPGAAGRXXXXXXXXXSGPPGPPGPAGKEGLRGPRGDQGPVG
RXXXXXXXXXXXXXXXXGPSGEPGTAGPPGTPGPQGLLGAPGFLGLPGSRGERGLPGVAGSVGEPGPL
GIAGPPGARGPPGNVGNPGVNGAPGEAGRDGNPGNDGPPGRDGQPGHKGERXXXXXXXXXXXXXXXXX
XXXXXXXXXHGSRGEPGPVGAVGPAGAVGPRGPSGPQGIRGDKGEPGDKGPRGLPGLKGHNGLQGLPG
LAGHHGDQGAPGAVGPAGPRGPAGPTGPAGKDGRTGQPGAVGPAGIRGSQGSQGPAGPPGPPGPPGPP
GPSGGGYDFGFDGDFYRA
```

Supplementary Fig. S2. Sequence coverages of the alpha 1 and 2 type I collagen helical regions (ref. *C. hircus* for COL1A1 and *O. aries* for COL1A2) of the impala. Grey indicates coverage of the reference sequence. Red indicates coverage of the impala-specific peptides.

>Alpha 1 chain collagen type I [*Aepyceros melampus*] 67% *C. hircus*

```
XXXXXXXXXSTGISVPGPMGPSGPRGLPGPPGAPGPQGFQGPPGEPGEPGASGPMGPRXXXXXXXXXX
XXXXXXXXXXXXXXXXXXXXXXXXXXXXXXXXXXXXXXGFSGLDGAKGDAGPAGPKGEPGSPGENGAP
GQMGPRXXXXXXXXXXXXXXXXXXXXXXXXXXXXXXXXXXXXXXXXXXXXXXXXXXXXXXXXXGSEGP
QGVRXXXXXXXXXXXXXXXXXXXXXXXXXXXGANGAPGIAGAPGFPGARGPSGPQGPSGPPGPKXXXX
XXXXXXXXXXXXXXGEPGPTGIQGPPGPAGEEGKRXXXGEPGPAGLPGPPGERXXXXXXXXXXXXXXX
XXXXXXXXXXXXXXXXXXGSPGEAGRPGEAGLPGAKGLTGSPGSPGPDGKTGPPGPAGQDGRPGPPGP
PGARGQAGVMGFPGPKXXXXXXXXXXXXGVPGPPGAVGPAGKDGEAGAQGPPGPAGPAGERGEQGPAG
SPGFQGLPGPAGPPGEAGKPGEQGVPGDLGAPGPSGARXXXXXXXXXGVQGPPGPAGPRXXXXXXXXX
XXXGDAGAPGAPGSQGAPGLQGMPGERXXXXXXXXXXXXXXXXXXXXXXXXXXXXXXGLTGPIGPPGP
AGAPGDKGETGPSGPAGPTGARXXXXXXXXXXXXXXXXXXXXXXXXXXXXXXXXXXXXXXXGDAGPPG
PAGPAGPPGPIGNVGAPGPKXXXGSAGPPGATGFPGAAGRVGPPGPSGNAGPPGPPGPAGKXXXXXXX
GETGPAGRPGEVGPPGPPGPAGEKGAPGADGPAGAPGTPGPQGIAGQRGVVGLPGQRXXXGFPGLPGP
SGEPGKQGPSGASGERGPPGPMGPPGLAGPPGESGRXXXXXXXXXXXXDGAPGAKGDRGETGPAGPPG
APGAPGAPGPVGPAGKSGDRGETGPAGPAGPIGPVGARXXXXXXXXXGDKGETGEQGDRXXXXXXGFS
GLQGPPGPPGSPGEQGPSGASGPAGPRGPPGSAGTPGKDGLNGLPGPIGPPGPRXXTGDAGPAGPPGP
PGPPGPPGPPSGGYDLSFLPQPPQEKXXXXXXXXXX
```

>Alpha 2 chain collagen type I chain [*Aepyceros melampus*] 52% *O. aries*

```
XXXXXXXXXXXXXXXXXXGPPGASGAPGPQGFQGPPGEPGEPGQTGPAGARXXXXXXXXXXXXXXXXX
XXXXXXXGVVGPQGARXXXXXXXXXXXXXXXXXXXXXXXXXXXXXXXXXXXXXXXXXXXXXXXXXXXX
XXXXXXXXXXXXXXXXXXXXXXXXXXXXXXXXXXXXXXXXXXXXXXXXXXXXXXXXXXXXXXXXXGEV
GLPGLSGPVGPPGNPGANGLPGAKXXXXXXXXXXXXXXXXXXGIPGPVGAAGATGARXXXXXXXXXXX
XXXXXXXXXXXXXXXXXXXXXXXXXXXXGSTGEIGPAGPPGPPGLRXXXXXXGLPGADGRAGVMGPAG
SRGATGPAGVRGPNGDSGRPGEPGLMGPRGFPGSPGNIGPAGKXXXXXXXXXXXXPGPIGPAGARGEP
GNIGFPGPKXXXXXXXXXXXXXXXXXXXXXXXXXXXXXXXXXXXXXXXXXXXXXXXXXXXXXXXXXXX
XXXXXXXXXXXXXXXXGIPGEFGLPGPAGARXXXGPPGESGAAGPAGPIGSRGPSGPPGPDGNKGEPG
VVGAPGTAGPSGPSGLPGERXXXXXXXXXXXXXXXXXXXXXXXXXXXXXXGAPGAVGAPGPAGANGDR
GEAGPAGPAGPAGPRGSPGERGEVGPAGPNGFAGPAGAAGQPGAKGERXXXGPKGENGPVGPTGPVGA
AGPSGPNGPPGPAGSRGDGGPPGATGFPGAAGRTGPPGPAGISGPPGPPGPAGKXXXXXXXXXXXXXX
XXXXXXXXXXXXXXXXXXXXXXXXXXXXXXXXXXXXXXXXXXXXXXXXXXXXXGLPGVAGSVGEPGPL
GIAGPPGARXXXXXXXXXXXXXXXXXXXXXXXXXXXXXXXXXXXXXXXXXXXXXXXXXXXXXXXXXXX
XXXXXXXXXXXXXXXXXXXXXXXXXXXXXXXGPSGPQGIRGDKXXXXXXXXXXXXXXXGHNGLQGLPG
LAGHHGDQGAPGAVGPAGPRGPAGPTGPAGKDGRTGQPGAVGPAGIRGSQGSQGPAGPPGPPGPPGPP
GPSGGGYDFGFDGDFYRX
```

Supplementary Figure S3. MS/MS spectra of distinguishing peptides of springbok and impala obtained as a result of *de novo* analyses of modern reference samples from the comparative anatomy collections of the Muséum national d'Histoire naturelle, Paris.

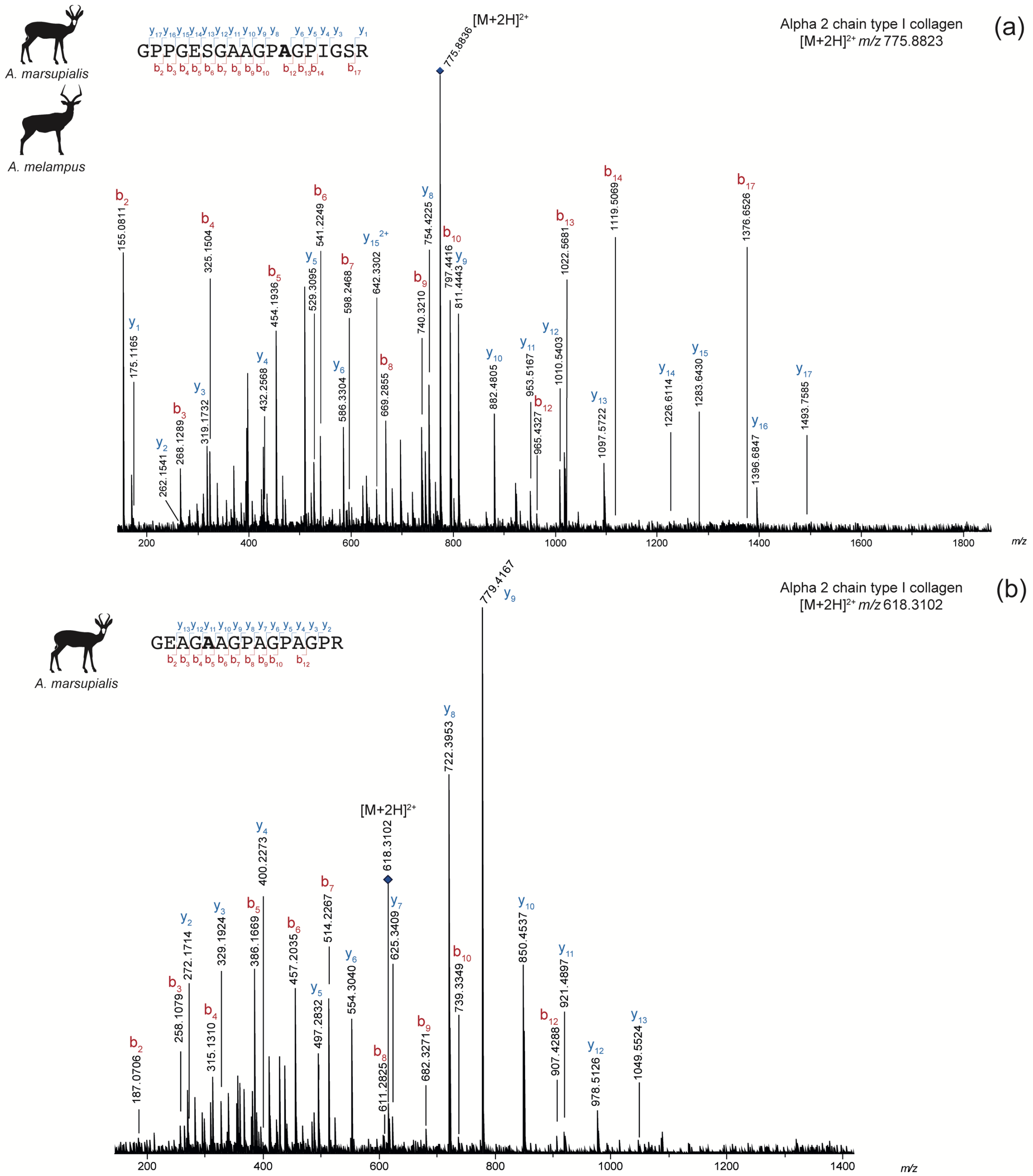

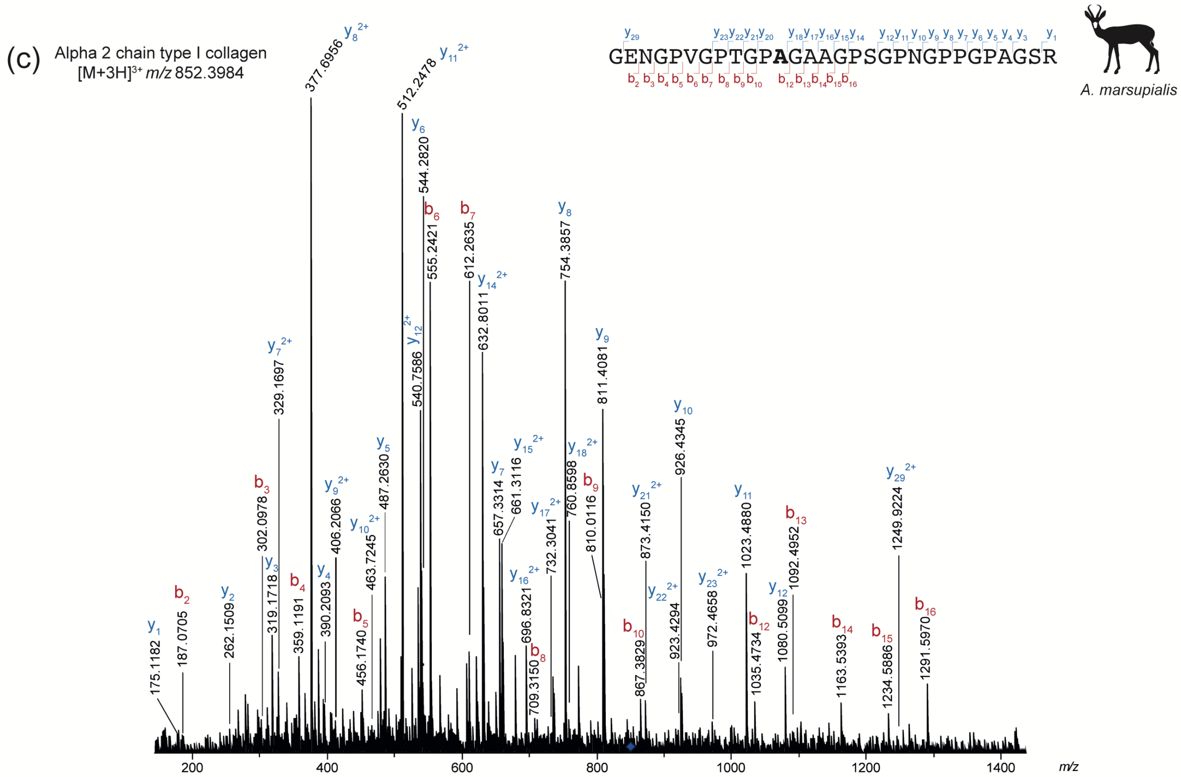
(c)
Alpha 2 chain type I collagen
[M+3H]3+ m/z 852.3984
GENGPVGPTGPAGAAGPSGPNGPPGPAGSR
A. marsupialis
175.1182
187.0705
262.1509
302.0978
319.1718
329.1697
359.1191
377.6956
390.2093
406.2066
456.1740
463.7245
487.2630
512.2478
540.7586
544.2820
555.2421
612.2635
632.8011
657.3314
661.3116
696.8321
709.3150
732.3041
754.3857
760.8598
810.0116
811.4081
867.3829
873.4150
923.4294
926.4345
972.4658
1023.4880
1035.4734
1080.5099
1092.4952
1163.5393
1234.5886
1249.9224
1291.5970
200
400
600
800
1000
1200
1400
m/z

Supplementary Figure S4. MS/MS spectra of distinguishing peptides for archaeological samples LC_113 (A); LC_149 (B); and LC_176 (C).

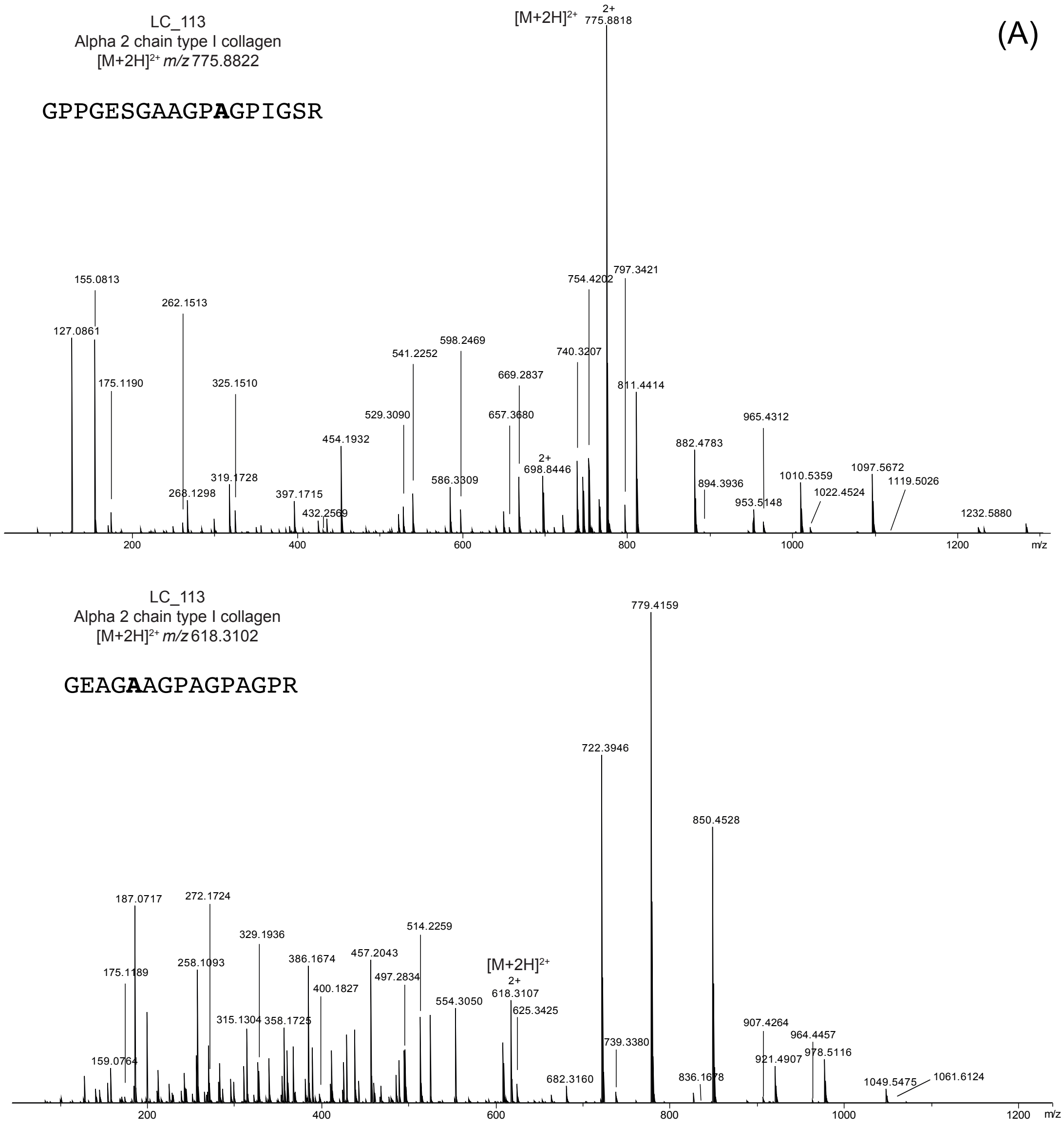

(B)

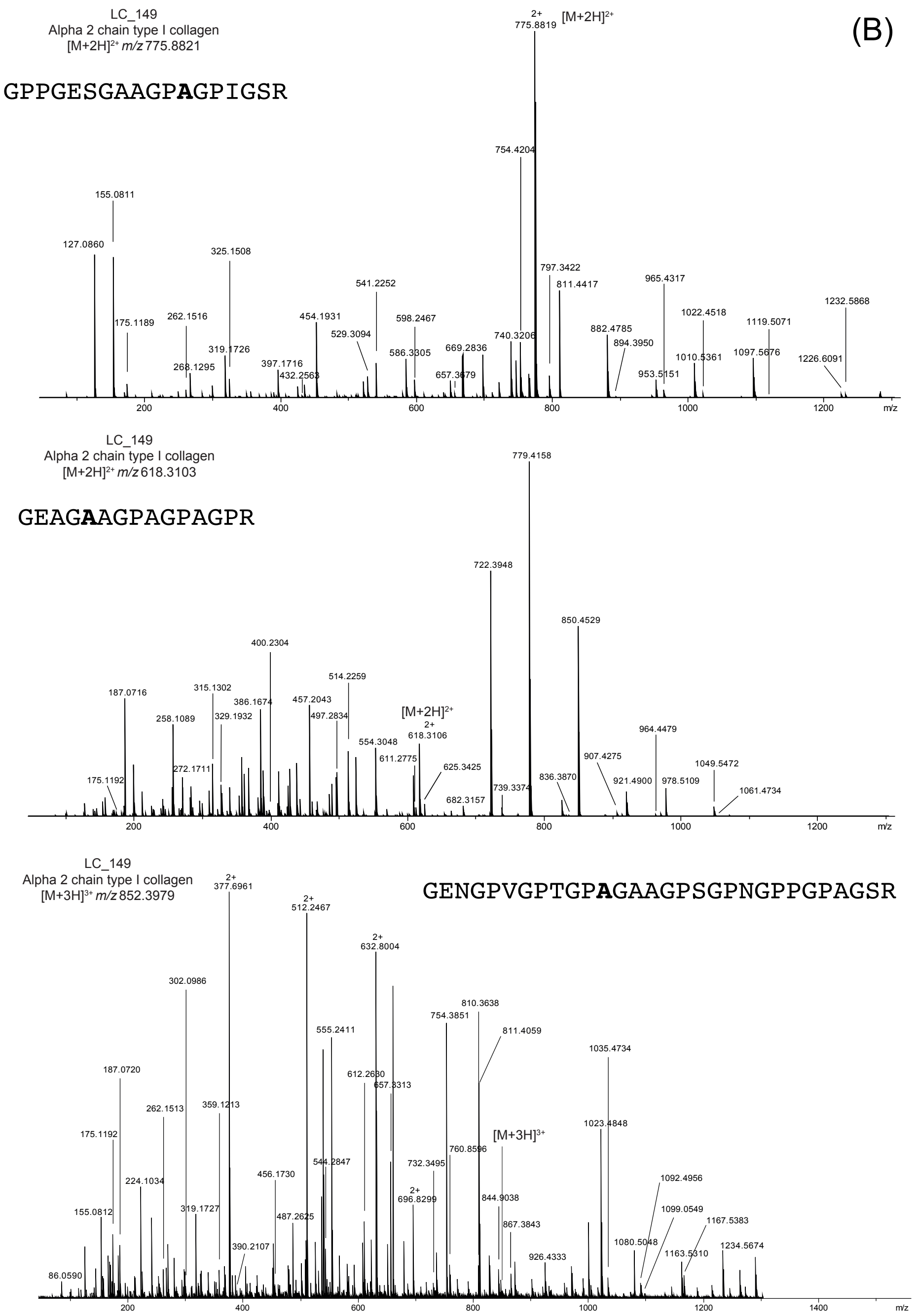
LC_149
Alpha 2 chain type I collagen
[M+2H]2+ m/z 775.8821
GPPGESGAAGPAGPIGSR
[M+2H]2+
LC_149
Alpha 2 chain type I collagen
[M+2H]2+ m/z 618.3103
GEAGAAGPAGPAGPR
[M+2H]2+
LC_149
Alpha 2 chain type I collagen
[M+3H]3+ m/z 852.3979
GENGPVGPTGPAGAAGPSGPNGPPGPAGSR
[M+3H]3+
m/z

(C)

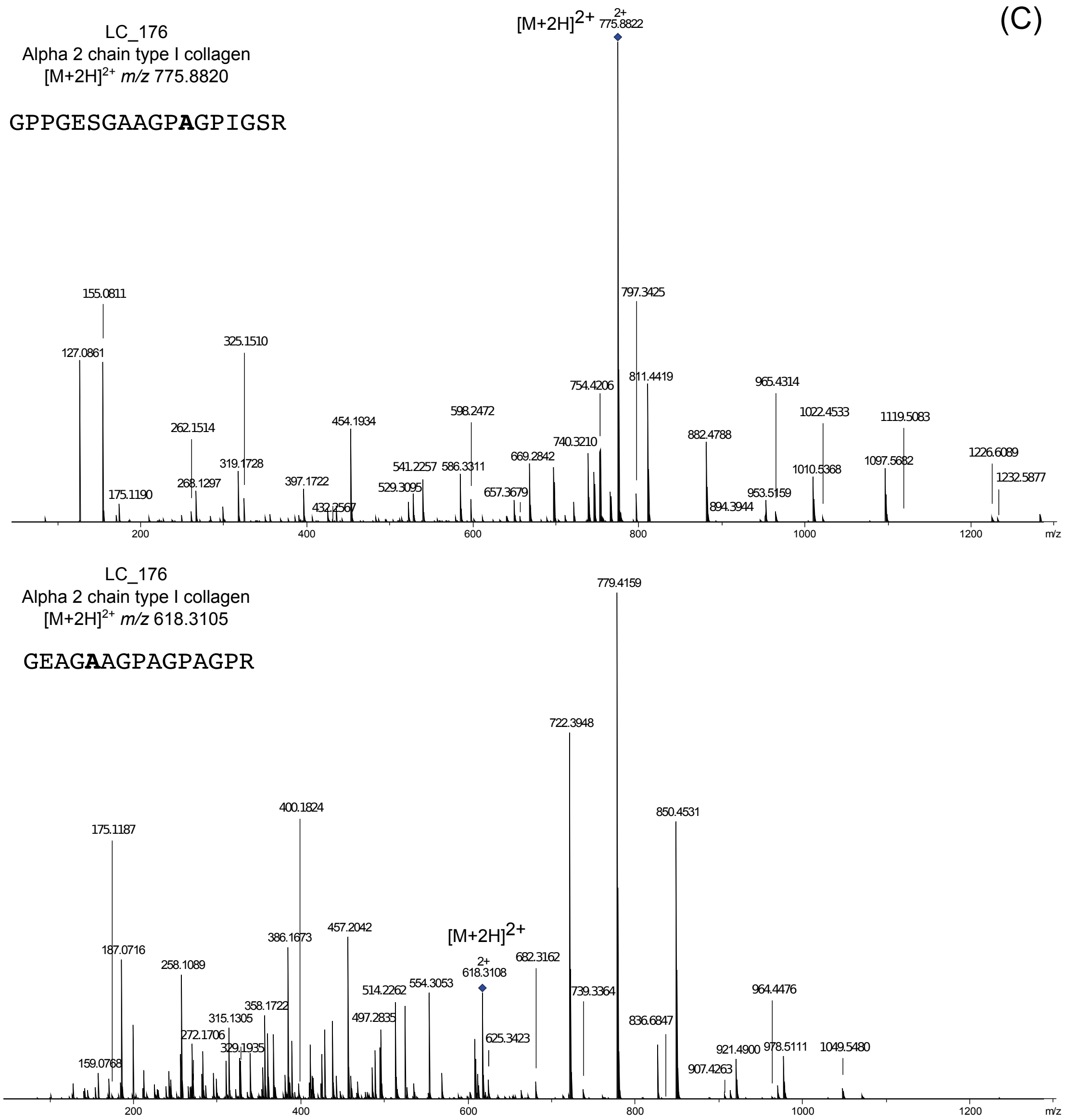
LC_176
Alpha 2 chain type I collagen
[M+2H]2+ m/z 775.8820
GPPGESGAAGPAGPIGSR
[M+2H]2+
2+ 775.8822
155.0811
127.0861
325.1510
262.1514
319.1728
268.1297
175.1190
454.1934
397.1722
432.2567
598.2472
541.2257
586.3311
529.3095
657.3679
669.2842
740.3210
754.4206
797.3425
811.4419
882.4788
894.3944
965.4314
953.5159
1022.4533
1010.5368
1119.5083
1097.5682
1226.6089
1232.5877
200
400
600
800
1000
1200
m/z
LC_176
Alpha 2 chain type I collagen
[M+2H]2+ m/z 618.3105
GEAGAAGPAGPAGPR
779.4159
722.3948
400.1824
175.1187
850.4531
457.2042
386.1673
187.0716
258.1089
[M+2H]2+
2+ 618.3108
682.3162
514.2262
554.3053
358.1722
739.3364
964.4476
315.1305
497.2835
272.1706
836.6847
329.1935
625.3423
921.4900
978.5111
1049.5480
159.0768
907.4263
200
400
600
800
1000
1200
m/z

Supplementary Table S6. Number of queries and of peptide spectral matches (PSMs) for each archaeological sample.

Sample code	Number of queries	Number of PSMs
LC_113	1515	240
LC_114	2167	213
LC_115	2279	284
LC_116	2406	845
LC_117	1932	617
LC_118	1938	603
LC_119	2153	703
LC_120	2380	786
LC_121	2316	814
LC_122	2404	834
LC_124	2223	723
LC_125	2252	765
LC_126	1910	550
LC_127	1916	452
LC_128	2345	774
LC_129	2161	790
LC_130	2283	758
LC_131	2238	826
LC_132	2052	562
LC_133	2258	737
LC_134	1979	496
LC_135	2367	829
LC_136	2094	522
LC_137	2561	780
LC_138	2398	834
LC_139	2209	698
LC_149	1469	233
LC_176	1590	264
